# Near-Linear Responsive and Wide-Range Pressure and Stretch Sensor Based on Hierarchical Graphene-Based Structures via Solvent-Free Preparation

**DOI:** 10.3390/polym12081814

**Published:** 2020-08-13

**Authors:** Jian Wang, Ryuki Suzuki, Kentaro Ogata, Takuto Nakamura, Aixue Dong, Wei Weng

**Affiliations:** 1 Center for Material Design Science, School of Integrated Design Engineering, Keio University, 3-14-1 Hiyoshi, Yokohama 223-8522, Japan; wangjian@keio.jp (J.W.); suzukiryuki@keio.jp (R.S.); kentaro2803@gmail.com (K.O.); takutonaka0406@gmail.com (T.N.); 2 College of Textile and Garment, Shaoxing University, Shaoxing 312000, Zhejiang, China; 3 State Key Laboratory for Modification of Chemical Fibers and Polymer Materials, College of Materials Science and Engineering, Donghua University, Shanghai 201620, China

**Keywords:** pressure sensor, stretch sensor, solvent-free, wearable devices, human motion detection

## Abstract

Flexible and wearable electronics have huge potential applications in human motion detection, human–computer interaction, and context identification, which have promoted the rapid development of flexible sensors. So far the sensor manufacturing techniques are complex and require a large number of organic solvents, which are harmful not only to human health but also to the environment. Here, we propose a facile solvent-free preparation toward a flexible pressure and stretch sensor based on a hierarchical layer of graphene nanoplates. The resulting sensor exhibits many merits, including near-linear response, low strain detection limits to 0.1%, large strain gauge factor up to 36.2, and excellent cyclic stability withstanding more than 1000 cycles. Besides, the sensor has an extraordinary pressure range as large as 700 kPa. Compared to most of the reported graphene-based sensors, this work uses a completely environmental-friendly method that does not contain any organic solvents. Moreover, the sensor can practically realize the delicate detection of human body activity, speech recognition, and handwriting recognition, demonstrating a huge potential for wearable sensors.

## 1. Introduction

In recent years, flexible electronic devices have attracted worldwide attention in both academic and industrial fields because of their excellent human-friendliness [1,2]. Flexible sensors, a primary member of flexible electronics, have already shown extensive applications, such as flexible strain sensors for measuring the body motion [3] and blood glucose sensors for measuring the health state of the human body so as to assist the medical staff in the care and diagnosis of patients [4,5]. In addition, flexible sensors can be used in software robots [6,7] human–computer interaction [8], and electronic skin [9,10]. The mechanism of flexible pressure and strain sensors is to convert the as-received external stimulus into an electrical signal. The conversions mainly include piezo-capacitance [11,12], piezoelectric [13], piezoresistive [14,15,16], and triboelectric processes [17,18]. Among them, piezoresistive flexible sensors are promising due to their simple structures [19,20], easy signal processing and collection, low costs, and easy manufacturing.

As to piezoresistive flexible sensors, conductive materials such as grapheme [21,22], carbon nanotubes (CNT) [23,24], metal nanowires [25,26,27], and conducting polymers [28,29,30] have been widely studied and used as sensing materials. For example, Wang et al. [31] designed a flexible stretch sensor by dissolving CNT in N, N-Dimethylformamide solution to form a uniformly mixed solution, which was then transferred to a rubber elastic belt pre-swelled by xylene melting. Shuai et al. [26] dispersed silver nanowires into a polydimethylsiloxane (PDMS) microarray film leading to a flexible pressure sensor with polyvinylidene fluoride (PVDF) dielectric layer. Wang et al. [32] selected multi-walled carbon nanotubes (MWCNTs) and PDMS diluted with toluene solution to prepare resistive tactile sensor arrays with coplanar electrodes that are capable of isolating sensing elements. In our previous work, a highly conductive film was prepared by dissolving graphene and Poly(3,4-ethylenedioxythiophene)-poly(styrenesulfonate)(PEDOT: PSS) in ethanol, on which a bionic, scaly PDMS film was formed resulting in a highly sensitive pressure sensor [33].

However, we should note that most processes for preparing the above-mentioned sensors require a large amount of organic solvents to assist the dispersion of conductive materials. In particular, some used toxic organic solvents containing benzene [31,32], which may lead to environmental problems and have potential hazards to human health. To this end, some solvent-free methods have been proposed. For example, Liao et al. [34] directly wrote a graphite layer on Xerox paper with a graphite pencil, and a graphite-based strain sensor was prepared. Recently, a new solvent-free printing ink has also been developed. Lee et al. [35] used aerodynamically focused nanoparticle (AFN) technology to directly print the ink to a substrate surface under the impact of inertia. Although these methods avoid the use of organic solvents, the prepared sensors had low sensitivity or failed to detect large strains, which limits the application and the promotion of solvent-free sensors.

Here, we constructed a new type of graphene hierarchical sensing layer, and the preparation process did not use any organic solvents; the resultant solvent-free graphene (SFG) sensor has excellent pressure and strain sensing characteristics. PDMS film was immersed directly into graphene nanoplate powders, and graphene nanoplates sufficiently adhered to the PDMS surface by the electrostatic force and van der Waals force between PDMS and graphene. Afterward, the uneven graphene nanoplates were flattened into a hierarchical layer under friction, effectively reducing the resistance and generating high sensing performance. The tensile strain detection limit of the SFG sensor is as low as 0.1%, and the gauge factor reaches 36.2 with excellent durability. Besides, the sensor has near-linear response and a large pressure response range up to 700 kPa. Practically, the sensor can not only detect the human body’s large strain activities, but also has excellent sensitivity to human blinking, speech recognition, and other small activities. This solvent-free method greatly reduces the production steps and costs, and has broad prospects in flexible sensors.

## 2. Experimental Section

### 2.1. Materials

Graphene nanoplatelets (MKCB3385, CAS No. 7782-42-5, SKU No. 900394, xGnP, C-300) were purchased from Sigma-Aldrich (Burlington, MA, USA). The surface area is 300 m^2^g^−1^, and the bulk density is between 0.2–0.4 g/cm^3^. PDMS (Sylgard 184, Dow Corning) was purchased from Tokyo Chemical Industry(Tokyo, Japan). Ag paste (DOTITE D-500) was purchased from Fujikura Kasei Co., Ltd., (Tokyo, Japan). In the future, biocompatible electro-conductive water-soluble glue can be used instead of this silver paste [36].

### 2.2. Fabrication of SFG Sensor

The base liquid and curing agent of PDMS were mixed in a weight ratio of 10:1, and bubbles in the mixture were extracted by a vacuum pump. Afterward, the liquid mixture was spin-coated onto a Polyethylene terephthalate(PET) film and then cured at 75 °C for 3 h. The as-obtained PDMS-coated PET film was completely immersed in graphene nanoplate powders, stood for 10 s, and then was taken out. In this process, a uniform but rough layer of graphene nanoplate powders adhered to the surface of the PDMS film. Next, a pressure of approximately 9 MPa given by an iron bar was placed on the graphene layer and moved horizontally back and forth to smooth the graphene layer. Finally, a PDMS film was spin-coated to cover the graphene layer. As for the sensing test, sensor samples were cut into strips with a width of 1 cm and a length of 5 cm. For each batch, there are 6 samples, among which there is little difference in sensing performance. Finally, copper wires were connected to the surface of the graphene layer at both ends using conductive silver paste.

### 2.3. Characterization and Measurements

Surface characteristics of the graphene layer were characterized by scanning electron microscope (SEM, Inspect F50, FEI Company, Hillsboro, OR, USA). Before the SEM test, the sample surface was sprayed with osmium to obtain a clear figure. The sensor’s resistance was measured and recorded by Keysight 34461A Digital Multimeter (Keysight Technologies, America) and ZM2372 LCR meter (NF Corporation, Yokohama, Japan). An IMADA force gauge was used to apply pressure to the sensor and record the values. Strain was measured using TRAPEZIUM LITE (Shimadzu Co.,Ltd., Kyoto, Japan).

## 3. Results and Discussion

**Figure 1:**
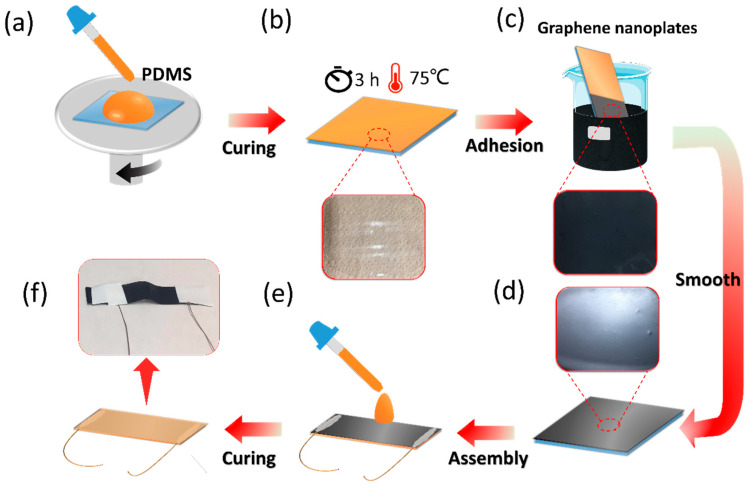
Preparation process and photo of the solvent-free graphene (SFG) sensor. (**a**–**e**) Schematic diagram of the preparation process of the SFG sensor. Partial enlargements in (**b**–**d**) are photos of different preparation steps of the SFG sensor. (**f**) Schematic and photograph of the SFG sensor.

The preparation process of the SFG sensor is shown in Figure 1. First, the PDMS solution was spin-coated at 350 rpm for 100 s (Figure 1a), then the spin-coated sample was placed in an oven at 75 °C for 3 h until PDMS was fully cured to form a clear PDMS film (Figure 1b). The PDMS film was directly immersed in a beaker containing graphene nanoplates, which automatically adhered to the surface of the PDMS film (Figure 1c). Then, after a simple smoothing action, a graphene film consisting of graphene nanoplates was formed with a smooth surface (Figure 1d). The prepared sample was cut to an appropriate size (1 cm × 5 cm), and copper wires were connected at both ends with conductive paste. To protect the sensor and increase the durability, a PDMS solution was coated on the graphene film and cured for packaging (Figure 1e). After that, the SFG sensor was achieved. Figure 1f shows the schematic and photograph of the SFG sensor. It should be noted that white tape was attached to both sides of the sensor to protect the junction of the copper wires and the sensor.

**Figure 2:**
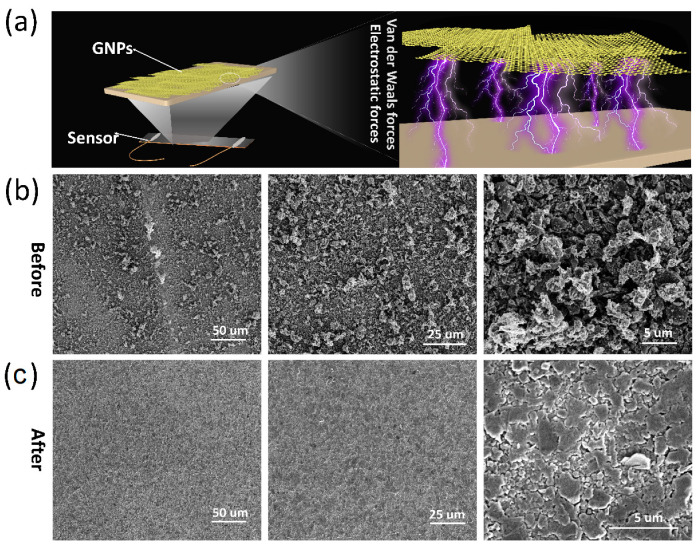
Surface morphology of the graphene layer of the SFG sensor. (**a**) A scheme of the SFG sensor based on stacked graphene nanoplates (GNPs). (**b**) Microscopic morphology of graphene nanoplates before smoothing. (**c**) Microscopic morphology of graphene nanoplates after smoothing.

Figure 2a shows the scheme of the SFG sensor. As shown in Figure 2a, the sensing layer is made of graphene nanoplates (GNPs), which are sandwiched by two PDMS films. Notably, PDMS has good bonding with graphene nanoplates by electrostatic force and van der Waals force. According to SEM images, the graphene nanoplates adhere to the surface of the PDMS film and exhibit granular structure with different sizes before smoothing (Figure 2b). This morphology is believed to be detrimental to the conductive path, making the resistance relatively large (Figure S1a) and affecting the sensitivity of the sensor. After the smoothing action, the graphene nanoplates stretched out to form a continuous and flat hierarchical graphene film (Figure 2b). Conventionally, graphene films have a sheet-like surface with solvent-assisted methods [37,38]. On the contrary, a relatively rough surface, which may be referred to as an isle-like surface, has been obtained in this experiment. More importantly, the smoothed structure makes the graphene nanoplates tighter to greatly reduce the resistance. After the smoothing action, the resistance of the graphene conductive layer decreased from 90.90 kΩ to 25.09 kΩ (Figure S1). Figure S2 shows a cross-sectional view of the graphene conductive layer in the SFG sensor. As can be seen from Figure S2a, the graphene nanoplates before smoothing are aggregated into uneven granules. After smoothing, a hierarchical graphene layer was formed with a thickness of 1.2 um and a flat surface, and the graphene nanoplates are stacked to form an excellent conductive path (Figure S2b). At the same time, it facilitates the coating of the subsequent PDMS protective layer. Therefore, the handy smoothing process provides a simple method for graphene nanoplates to construct high-performance sensors.

**Figure 3:**
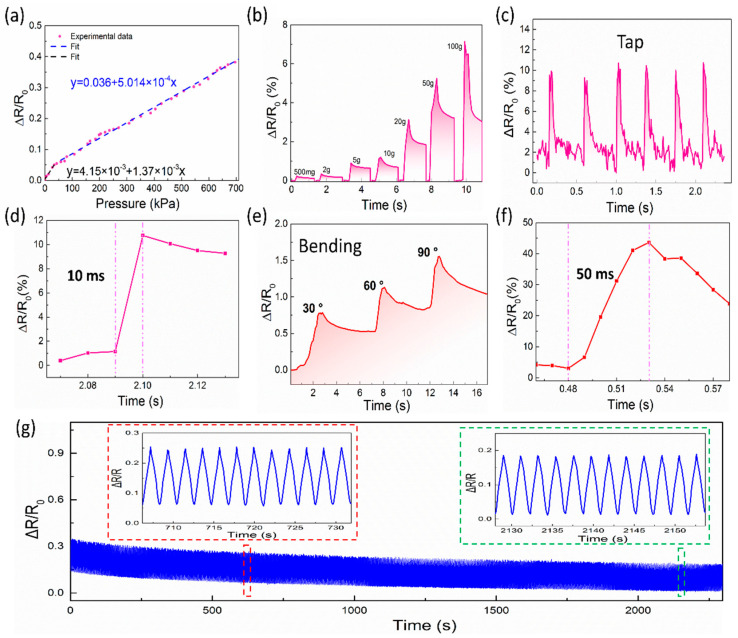
(**a**) Relative resistance change vs. pressure curves of the SFG sensor. (**b**) Resistance response of the SFG sensor with different weights. (**c**) Resistance response curve upon the finger continuously tapping the sensor surface. (**d**) The transient response time of the finger tapping the SFG sensor. (**e**) Resistance response curve of the SFG sensor under different bending angles. (**f**) The response time under bending. (**g**) Repetitive characteristics of the SFG sensor over 1000 bending cycles. Inset: enlarged resistance change curves at different bending cycles.

In order to evaluate the pressure sensing performance of the SFG sensor, the sensor was connected to the electrical sensing analysis device through conductive copper wires. Then, a real-time pressure sensing experiment was performed on a dynamometer platform. The pressure sensitivity of the sensor is defined as S = δ(∆R/R_0_)/δP, where P is the externally applied pressure, ∆R is the relative change in resistance, and R_0_ is the initial resistance without external pressure [39]. Figure 3a shows a typical resistance diagram at different external pressures. As shown in Figure 3a, the relationship between P and ΔR/R_0_ can be divided into two parts in terms of pressure range: low-pressure range (0 kPa to 50 kPa) and high-pressure range (50 kPa to 700 kPa). An enlarged view of Figure 3a, from 0 kPa to 100 kPa, is shown in Figure S3. It is worth noting that the sensor exhibits an excellent wide-range pressure response and high linearity in a large pressure range. In the low-pressure range, the sensitivity of the SFG sensor is 1.37 × 10^−3^ kPa^−1^. In the high-pressure range, the sensitivity is 5.014 × 10^−4^ kPa^−1^. The difference between sensitivities at different pressure ranges is an important indicator to evaluate the quality of sensors. By comparison with other sensors (Figure S4 and Table S1), the SFG sensor exhibits a near-linear response in an ultra-wide pressure window, showing excellent sensitivity stability. Therefore, the SFG sensor can be used to detect different pressure levels with high sensitivity over a wide pressure range as large as 700 kPa. To be mentioned, the minimum resolution of the used IMADA force gauge is 0.1 N, which is converted into a pressure value of 1 kPa. Although the lower limit of IMADA force gauge is 1 kPa, the lower limit of the SFG sensor is smaller than 1 kPa, which will be verified in the following part by using weights.

Applications with pressures below 10 kPa include tactile sensing, electronic skin, medical diagnostics, etc., while larger pressure responses can be used for weight sensing [40]. Figure 3b shows the resistance response curve of the SFG sensor upon different weights. As the weight increases, the instantaneous relative resistance increases significantly. It is also noted that as the static pressure time increases, its relative resistance decreases. This is because the sensing layer of the sensor is composed of graphene nanoplates without any chemical bondings between each other. When the sensor is momentarily exposed to pressure change, the PDMS layer squeezes the graphene nanoplates to generate a rapid change resulting in the quick response of the sensor. Under the relatively long static pressure, the PDMS layer and the graphene layer slowly shift and recover inside, which makes the relative resistance decrease.The minimal weight used here is 500 mg, which equals about 55 Pa when divided by the contact area of the weight. Obviously, 55 Pa is much smaller than the lower limit of the IMADA force gauge, showing the excellent sensitivity of the SFG sensor. Subsequently, the response of the SFG sensor under dynamic pressure was further studied. The relative resistance curve of the finger continuous tap is shown in Figure 3c. The relative resistance of the SFG sensor exhibits a continuous dynamic response when successively tapping the sensor. After the finger is released from the sensor, the relative resistance quickly returns to the initial state. In addition, Figure 3d shows the response time (10 ms) of the SFG sensor to the tap, thus ensuring a sensing response to the transient pressure. Based on this function, the SFG sensor is expected to be applied to real-time pressure measurements under dynamic stimulation. To explore the potential application of the sensor in flexible electronics, the bending performance of this sensor was studied. Figure 3e shows the relative resistance response to its bending deformation angle. At different bending angles, the sensor exhibits a significant relative resistance response. Furthermore, the bending response time is 50 ms, showing excellent bending response. In addition, long-term reliability is one important indicator for sensors in actual use. Therefore, the repeatability of the sensor was tested, as shown in Figure 3g. Under continuous testing of 1000 bending cycles, there is no significant instability or fault in the relative resistance change of the sensor. It can be seen in the magnification curves (inset) that the sensor maintains high stability and repeatability over different time periods, which are critical for practical applications. These results imply that the SFG sensor offers a distinct possibility for dynamic human motion monitoring.

**Figure 4:**
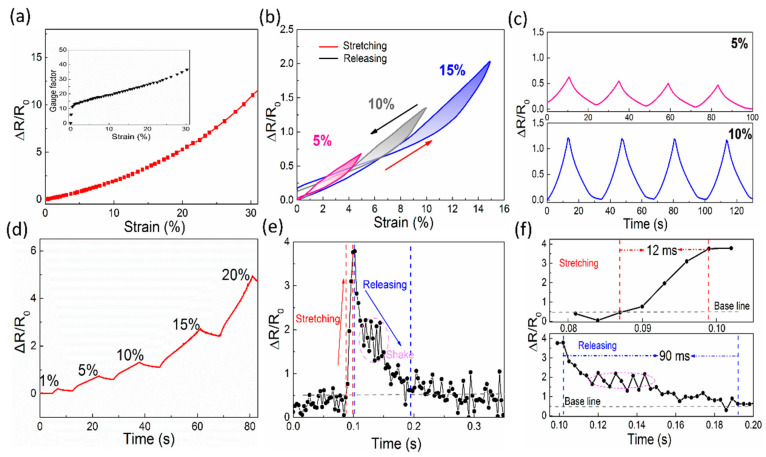
Performance of the SFG sensor for strain sensing. (**a**) Relative resistance changes of the SFG sensor versus the applied strain. Inset: gauge factor (GF) of the SFG sensor at different strains. (**b**) Relative resistance change of the SFG sensor during load/unload cycles. (**c**) Relative resistance response of the SFG sensor during repeated load/unload cycles at 5% and 10% strains. (**d**) A stepwise curve of relative resistance change versus time for the SFG under different strains. (**e**) Relative resistance change under fast stretching. (**f**) Response time and recovery time of the SFG sensor under stretching.

Besides excellent pressure sensing performance, the SFG sensor also shows excellent strain sensitivity. Figure 4a shows the typical resistance variation curve of the SFG sensor during stretching. The relative resistance of the sensor increases as the applied strain increases. The sensitivity of the strain sensor can be expressed by gauge factor (GF), which can be calculated by the following formula: GF = (∆R/R_0_)/ε, where R_0_ and ε represent the initial resistance and the applied strain, respectively [41]. During the stretching process of the sensor, the SFG sensor can show an excellent positive linear resistance change within the 30% strain range, and the maximum GF is 36.2, which is equivalent to the newly reported printed strain sensor [42]. Besides, the SFG sensor is capable of measuring tensile strain within 30%, which makes it suitable for monitoring the subtle movement of the human body than conventional metal-based sensors. To check the repeatability, repeated stretch/release cycles were applied to the sensor. Figure 4b shows the relative resistance response of the sensor as it is stretched to 5%, 10%, and 15%. The results show that the SFG sensor can almost return to the initial resistance when the stress is released, and the strain hysteresis of the residual portion is negligible, relative to the total strain. Meanwhile, the repeatability of the sensor under two different strains was also studied, as shown in Figure 4c. The relative resistance jumps rapidly when the sensor is stretched and recovers after release, thus presenting a repeatable response to each strain load. As shown in Figure 4d, the curve of the relative resistance versus time under stepwise stretching from 1% to 20% indicates that the signal can respond to different stretching deformations with corresponding relative resistance. Figure 4e shows the response curve of the sensor during rapid stretching. From its partial enlargement (Figure 4f), it is shown to have an excellent response time of 12 ms and a recovery time of 90 ms. The ultra-fast response time is due to the rapid slippage of the graphene nanoplates during stretching, resulting in significant changes in relative resistance, which is fast enough for medical applications.

**Figure 5:**
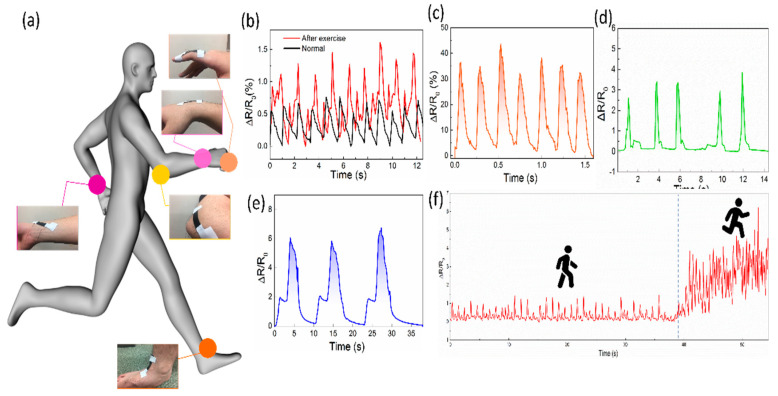
Monitoring various human motions using the SFG sensor. (**a**) A schematic diagram of the SFG sensor connected to different locations of the human body to monitor the subtle movements of the human. (**b**) Arterial pulse waves of an adult male measured under normal and exercise conditions, respectively. (**c**) Resistance responses to repetitive finger-bending motion. (**d**) Signals of the SFG sensor mounted on (**d**) wrist joint and (**e**) elbow joint. (**f**) Signals of the SFG sensor mounted on ankle monitoring walking and jogging.

Thanks to its excellent high sensitivity, wide working range, and excellent stability, the SFG sensor can be applied directly to the surface of human skin to monitor human body pressure, bending, and stretching deformation. Figure 5a shows an overview of the sensor at different locations on the human skin, indicating that the sensor has excellent skin-friendly and wearable properties. The wrist pulse index is a key physiological signal for providing arterial blood pressure and heart rate measures, which can provide an important reference for human health. For example, cardiovascular disease is detected based on arterial blood pressure of the wrist pulse. The SFG sensor is applied to the position of the wrist near the artery for monitoring the pulse signal. Figure 5b shows the pulse signal of a male adult volunteer. The results in Figure 5b show that the sensor can not only detect obvious pulse pressure signals but also distinguish small pulses according to different states of the body. At rest, the male volunteer had a pulse rate of 55 beats per minute. It should be noted that in most cases, the pulse rate of healthy adult males is considered to be between 60 and 100 beats per minute. However, according to some scholars, the pulse rate of adult males could be from 50 to 90 beats per minute, this result is consistent with healthy adults [43]. Under normal circumstances, in the magnified image of the pulse signal as shown in Figure S5, three subtle peaks P1, P2 and P3 can be seen, representing the peak pressure during the early systole, the peak pressure during the systole and the peak pressure during the diastolic phase [44]. After physical exercise, the wrist pulse frequency increased to 90 times/minute, and the relative resistance signal strength was significantly higher than the quiet state. These results indicate that the SFG sensor can detect subtle pressure changes and has great potential in wearable diagnostic equipments. Besides, the English alphabet can be written directly on the sensor to identify signal changes for different alphabets. Figure S6 shows the change in relative resistance of different alphabets written on the SFG sensor, such as “K”, “E”, “I” and “O”. By attaching the sensor to the finger, the bending of the finger can be detected. When the finger is bent inward, the pressure is applied to the sensor, and after the finger recovers, the pressure applied to the sensor disappears, and the relative resistance signal of the sensor also returns to the initial state. Figure 5c shows the relative resistance response of the sensor during repeated bending-recovery of the finger, indicating that the sensor can be effectively used to monitor the state of the finger.

When the wrist is bent, its skin is not only subjected to stretching and bending but also affected by the joint’s pressure on the bending. The SFG sensor was mounted on the outer surface of the wrist and its sensing performance was measured. During the bending of the wrist, the sensor is subjected to tensile deformation, which causes the relative resistance of the sensor to increase rapidly. When the wrist moves to the initial position, its relative resistance also returns to the initial state. In addition, the SFG sensor exhibits stable and repeatable sensing performance during the repeated bending/relaxation of the wrist. The SFG sensor was attached to the joint position of the elbow, its response to changes in the elbow was examined, and the change in relative resistance was recorded in real-time. As shown in Figure 5e, during the bending of the elbow, two peaks can be seen. The reason for this phenomenon is that when the elbow starts to move, the bending deformation of the sensor is mainly subjected to the stretching force, showing a small peak. When the bending of the elbow is increased, the sensor is subjected to both stretching and joint pressure, and its relative resistance exhibits a large peak. When the elbow returns to its initial state, its sensing signal also quickly returns to its initial value. As shown in Figure 5f, the SFG sensor is attached to the ankle and collects signals for walking and jogging, showing excellent repeatability and gait recognition. The SFG sensor can accurately distinguish the lifting and landing of each movement according to different motion states, thereby further identifying the motion.

**Figure 6:**
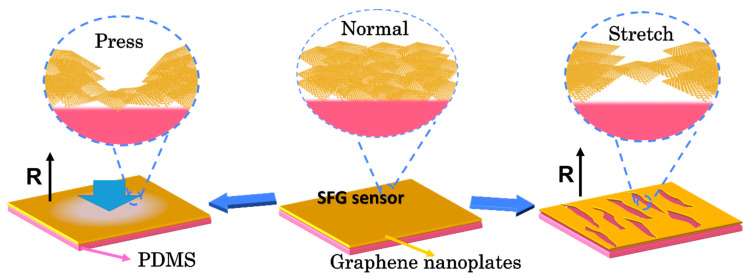
A schematic model of the response mechanisms of the SFG sensor.

In order to understand the working principle of the sensor, a simple model was developed and the response mechanisms of the SFG sensor were analyzed (Figure 6). Firstly, the mechanism of the SFG sensor as a pressure sensor was analyzed. As shown in Figure S7, when forming the outer PDMS protective film, the flowable PDMS liquid can partially penetrate into the gaps between graphene nanoplates. After solidification, PDMS is bonded with some graphene nanoplates to form the upper graphene layer. In addition, some graphene nanoplates are self-stacked as the middle graphene layer. Furthermore, some graphene nanoplates adhere to the surface of the bottom PDMS film, which is the bottom graphene layer. Therefore, there are three layers of graphene nanoplates in total. When the SFG sensor is under pressure, the upper graphene layer is significantly compressed and jostles the middle graphene layer resulting in the resistance increase. Next, the tensile strain sensing mechanism of the SFG sensor was analyzed. When the SFG sensor is longitudinally stretched, the upper graphene layer closely attached to the PDMS film forms cracks, and at the same time, the PDMS film shrinks laterally, so that the area of the cracks is relatively reduced. Additionally, the middle graphene layer can move to these cracks so that the resistance change is relatively small at this time. As the tensile deformation increases, the number of transverse cracks and the crack gap increase significantly, while the free graphene nanoplates in the middle graphene layer are not enough to fill all the cracks, increasing the relative resistance of the sensor. With this multi-layered graphene hierarchical structure, the SFG sensor achieves sensitive pressure and stretch responses.

## 4. Conclusions

We designed a solvent-free preparation strategy and successfully prepared a graphene-based sensor with excellent pressure and strain responses. This sensor was fabricated without any organic solvents, using only the strong electrostatic and van der Waals forces between graphene nanoplates and PDMS. The sensor exhibits a wide pressure range (up to 700 kPa), a small strain detection limit (0.1%), a large GF of 36.2, and a short response time. Moreover, the sensor shows near-linear responsiveness and excellent stability and durability. Practically, the sensor can be directly attached on the skin for monitoring local and subtle movements of the human body, such as finger bending, joint bending, pulse, and handwritten text recognition. This facile and effective strategy is probably extended to other conductive materials to devise high-performance flexible sensors for monitoring human physiological signals, software robots, and human–machine interfaces.

## Supplementary Materials

The following are available online at https://www.mdpi.com/2073-4360/12/8/1814/s1. The following files are available free of charge. Supplementary (PDF), Video S1 (AVI). Figure S1: Photos of resistance measurement of the graphene layer before and after smoothing, Table S1: Comparison of SFG sensor and other pressure sensors, Video S1: Bending durability of SFG sensor.
