# Supplementary material for: Near-Linear Responsive and Wide-Range Pressure and Stretch Sensor Based on Hierarchical Graphene-Based Structures via Solvent-Free Preparation
Source: Polymers (Basel). 2020 Aug 13;12(8):1814. doi: 10.3390/polym12081814 (PMC7465154; doi:10.3390/polym12081814)
Supplement: Supplementary file 1 [file polymers-12-01814-s001.pdf]

Supporting Information

Near-linear responsive and wide-range pressure and stretch sensor based on hierarchical graphene-based structures via solvent-free preparation

Jian Wang[ab], Ryuki Suzuki[a], Kentaro Ogata[a], Takuto Nakamura[a], Aixue Dong[b*] and Wei Weng[a,c*]

[a.] *Center for Material Design Science, School of Integrated Design Engineering, Keio University, 3-14-1 Hiyoshi, Yokohama 223-8522, Japan*

[b.] *College of Textile and Garment, Shaoxing University, Shaoxing, 312000, Zhejiang, China*

[c.] *State Key Laboratory for Modification of Chemical Fibers and Polymer MaterialsCollege of Materials Science and Engineering, Donghua University, Shanghai 201620, China*

Corresponding Author

* *jwangjn@gmail.com; wengwei@dhu.edu.cn*

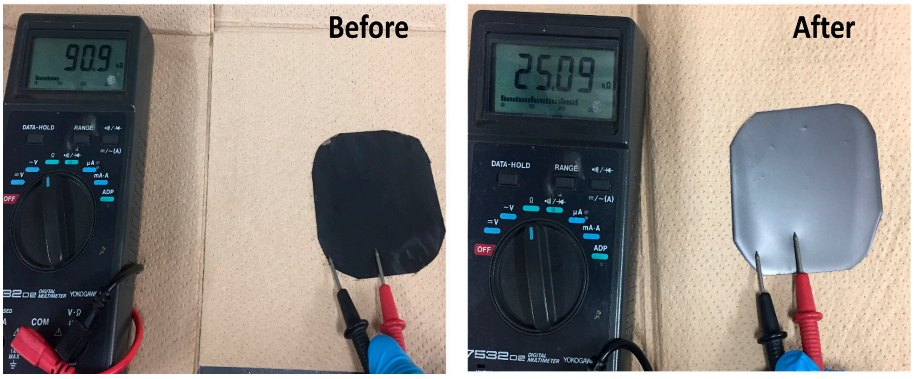

Figure S1. Photos of resistance measurement of the graphene layer before and after smoothing~~wiping~~.

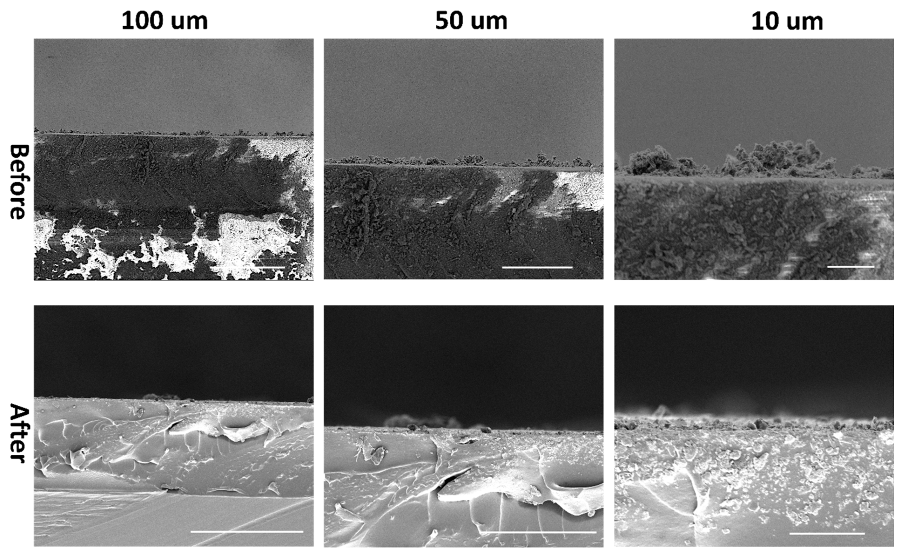

Figure S2. Cross-sectional SEM images of ~~cross section of~~the –graphene layer before and after smoothing.

Formatted: Font: Times New Roman, 12 pt, English (United Kingdom)

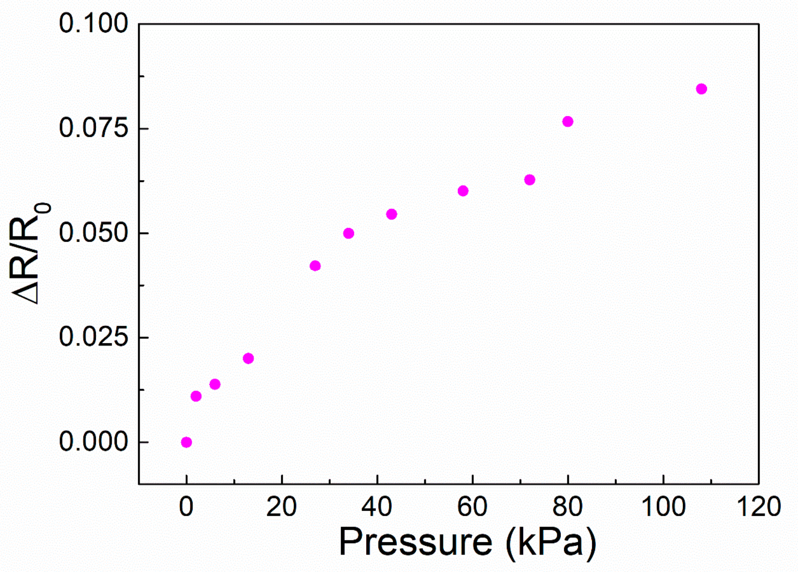

wiping

Figure S3. Relative resistance change vs. pressure curves of the SFG sensor in a pressure range of 0 – 100 kPa.

Formatted: Centered

Formatted: Font: Not Bold

Formatted: Font: Not Bold

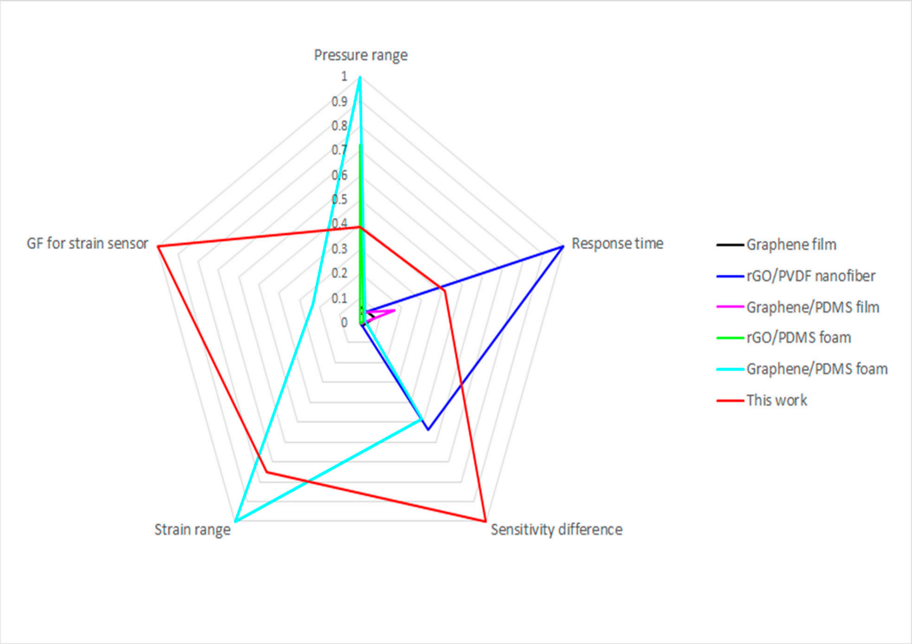

Figure ~~S2~~S4. Radar charts~~plots~~ comparing sensing performance between the SFG sensor and some related~~with~~ graphene-based~~other~~ sensors.[1–5]

TableS1 Comparison of SFG sensor and other pressure sensors

Sample	Materials	Methods	Pressure sensitivity	Pressure response time	Strain Sensitivity (GF)	Strain response time	Solvent
1[6]	SEBS/AgNPs	Electrospinning (THF/DMF)	71.07kPa^{-1} (0.2~60 Pa); 7.38 kPa^{-1} (63~95 Pa)	<2 ms	/	/	DMF/THF/Ethanol
2[7]	MXenes/ tissue paper/ polylactic acid	Coating	0.55kPa^{-1} (23~982 Pa), 2.52 kPa^{-1} (10~30kPa)	11 ms	/	/	LiF/HCl
3[8]	MXene/textile	Dip-coating	12.095 kPa^{-1} (29–40kPa); 3.844 kPa^{-1} (<29 kPa)	26 ms	/	/	HF/ Ethyl alcohol
4[9]	Carbon nanotube/ Ni-coated cotton yarn/ Polyurethane	Electrospinning	16.52 N^{-1} (0.003–5N)	0.03 s	/	/	DMF/ THF/ Ethyl alcohol
5[10]	Silver nanowires /ZnO nanocrystals (NCs)	Chemical treatment	3.23 × 10^{3} kPa^{-1} (<10kPa); 3.46 × 10^{2} kPa^{-1} (10~75kPa)	120ms	/	/	Toluene /Benzyl ether /Ethanol /Octadecanol
6[11]	PDMS / PET/ITO	Triboelectric nanogenerators	0.136 kPa^{-1}(<110kPa)	< 9.9 ms	/	/	IPA
7[12]	SWCNTs/PDMS	Membrane transfer	0.59kPa^{-1}· (~124 kPa)	/	0.68 (~300%)	/	Chloroform

8[13]	Nylon fiber/AgNWs/PDMS/Carbon black	Dip-coating	4.29 N^{-1} (0~0.2 N) 0.02 N^{-1} (0.2~2N)	8ms	(~100%)	/	Glycerol/Ethanol/Acetone/Chloroform
9[14]	Poly(octamethylene maleate (anhydride) citrate) (POMaC)/ Poly(glycerol sebacate) (PGS)/ Polylactic acid	Benchtop process	0.7 ± 0.4 kPa−1 (< 1 kPa); 0.13 ± 0.03 kPa−1 (5~10 kPa	Millisecond range	0.5 (0–15%)	Millisecond range	1,8-octanediol/ Ethyl acetate
10[15]	Yarn/AgNW solution	Dip-coating	0.096 kPa^{-1} (< 0.1kPa) 1.1 MPa^{-1}(10 ~50 kPa)	32ms	3.2(<50%)	70ms	Ethylene glycol
11[16]	Silver Nanoparticle/ Polyimide (PI) film, PET	Aerodynamically Focused Nanoparticle	/	/	14 (0.2~1%)	/	None
12[17]	MWCNTs,/Polyimide tape	Adhesive	0.549 kPa^{-1} (<100kPa)	<32ms	/	/	None
13[18]	Indium Tin Oxide /PDMS	Sputter	0.21 kPa^{-1} (0~30Pa) 1.91kPa^{-1} (30~70kPa)	/	4000 (2%)	1ms	None
14[19]	Graphite/paper	Mechanical drawing	4.77%/kPa (<30kPa)	0.3ms	/	/	None
15[20]	Cellulose/Paper/Graphene	Mechanical drawing	0.003kPa^{-1} (<45kPa)	/	/	/	None

This work	Graphene nanosheets, PDMS	Electrostatic adsorption	1.37×10^{-3} kPa^{-1} (0 kPa to 50 kPa); 5.014×10^{-4} kPa^{-1} (50 kPa to 700 kPa)	10ms	36.2 (~30%)	12ms	None

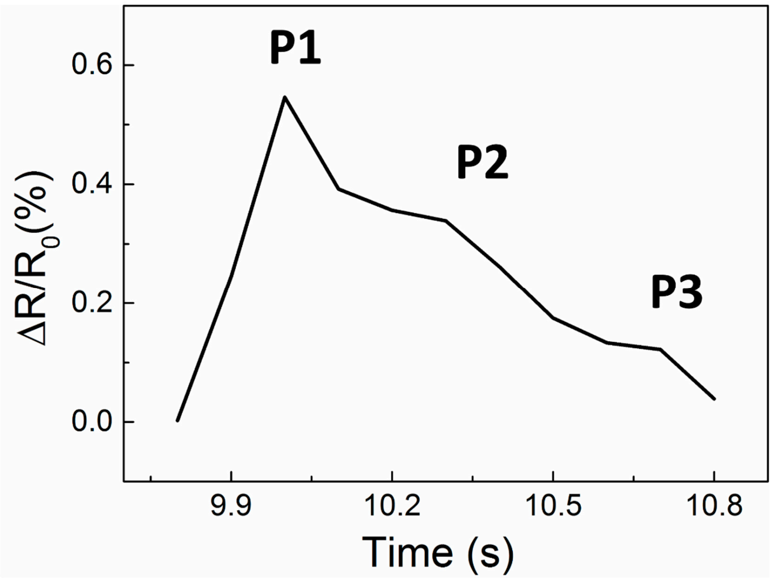

Figure S54. Waveform of one sSingle pulse signal. waveform

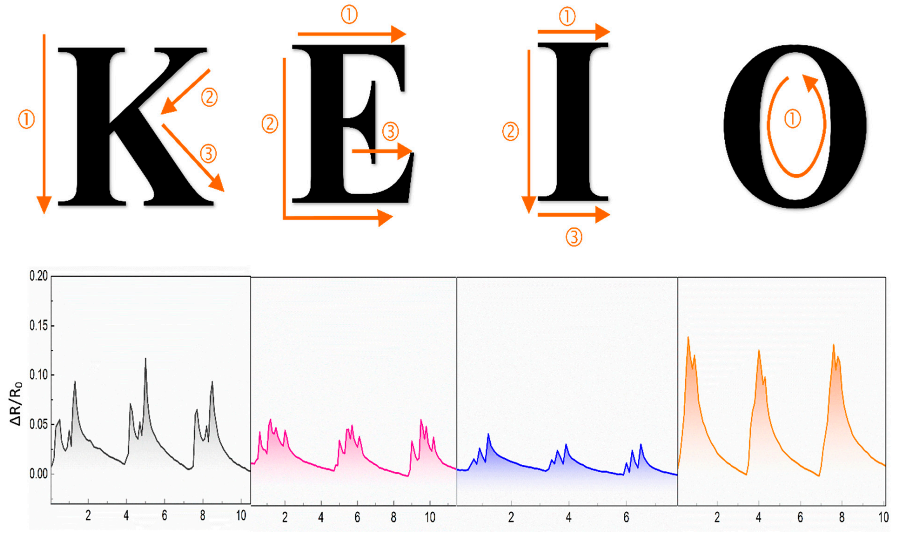

Figure S6~~5~~. Responsive s~~S~~ignal ~~response ~~diagrams for writing English letters on the SFG sensor.

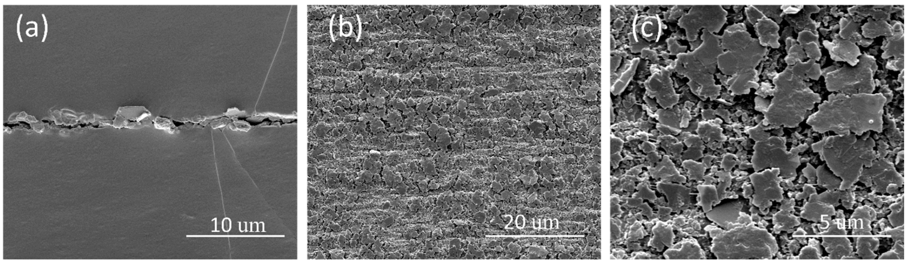

Figure S7~~6~~. (a) C~~The c~~ross-sectional view of a SFG~~a~~ sensor~~ incorporating a PDMS protective layer~~. (b,c) ~~The ~~SEM images~~diagram~~ of a~~the~~ stretched SFG sensor ~~stretched before coating~~without PDMS protective film.

References for Supporting Information

(1) Tian, H.; Shu, Y.; Wang, X.-F.; Mohammad, M. A.; Bie, Z.; Xie, Q.-Y.; Li, C.; Mi, W.-T.; Yang, Y.; Ren, T.-L. A Graphene-Based Resistive Pressure Sensor with Record-High Sensitivity in a Wide Pressure Range. *Sci Rep* **2015**, *5* (1), 8603.

(2) Lou, Z.; Chen, S.; Wang, L.; Jiang, K.; Shen, G. An Ultra-Sensitive and Rapid Response Speed Graphene Pressure Sensors for Electronic Skin and Health Monitoring. *Nano Energy* **2016**, *23*, 7–14.

(3) Xia, K.; Wang, C.; Jian, M.; Wang, Q.; Zhang, Y. CVD Growth of Fingerprint-like Patterned 3D Graphene Film for an Ultrasensitive Pressure Sensor. *Nano Res.* **2018**, *11* (2), 1124–1134.

(4) Pang, Y.; Tian, H.; Tao, L.; Li, Y.; Wang, X.; Deng, N.; Yang, Y.; Ren, T.-L. Flexible, Highly Sensitive, and Wearable Pressure and Strain Sensors with Graphene Porous Network Structure. *ACS Appl. Mater. Interfaces* **2016**, *8* (40), 26458–26462.

(5) Samad, Y. A.; Li, Y.; Alhassan, S. M.; Liao, K. Novel Graphene Foam Composite with Adjustable Sensitivity for Sensor Applications. *ACS Appl. Mater. Interfaces* **2015**, *7* (17), 9195–9202.
